# SOCIAL ISOLATION AND SUBJECTIVE WELL-BEING AMONG OLDER ADULTS: AN EXAMINATION BY RACE/ETHNICITY AND GENDER

**DOI:** 10.1093/geroni/igae098.2543

**Published:** 2024-12-31

**Authors:** Nhan Nguyen, Zhiyong Lin

**Affiliations:** The University of Texas San Antonio, San Antonio, Texas, United States; The University of Texas San Antonio, San Antonio, Texas, United States

## Abstract

Social isolation has emerged as a significant social determinant of health in later life. However, our understanding of how this link may vary across race/ethnicity and gender remains limited. To address this gap, we utilized longitudinal data from the Health and Retirement Study (2008-2020) to investigate the association between social isolation and subjective well-being (life satisfaction, positive/negative affection) among older adults. We examined variations in this relationship across race/ethnicity and gender subgroups. Our results demonstrate that social isolation is consistently linked to reduced subjective well-being for older adults, irrespective of race/ethnicity and gender. However, we observed nuances in this association among Hispanic older adults. Specifically, while both White and Hispanic older adults exhibit higher subjective well-being when they are not or rarely isolated compared to Black individuals and their isolated counterparts, among socially isolated older adults, particularly isolated men, Hispanic individuals tend to report higher levels of subjective well-being compared to their White and Black counterparts. Moreover, significant gender disparities in subjective well-being were identified among socially isolated older adults across all racial/ethnic groups, with women experiencing greater disadvantage compared to men. We also found that economic resources and health-related factors mediate the relationship between social isolation and subjective well-being. This study underscores the critical importance of addressing social isolation as a potential risk factor for mental health among older adults, especially those in vulnerable groups. It emphasizes the necessity for targeted interventions aimed at reducing social isolation and promoting social engagement within diverse communities to mitigate mental health disparities.

